# Target 5000: Target Capture Sequencing for Inherited Retinal Degenerations

**DOI:** 10.3390/genes8110304

**Published:** 2017-11-03

**Authors:** Adrian Dockery, Kirk Stephenson, David Keegan, Niamh Wynne, Giuliana Silvestri, Peter Humphries, Paul F. Kenna, Matthew Carrigan, G. Jane Farrar

**Affiliations:** 1 The School of Genetics & Microbiology, Trinity College Dublin, Dublin 2, Ireland; peter.humphries@tcd.ie (P.H.); paul.kenna@tcd.ie (P.F.K.); carrigma@tcd.ie (M.C.); jane.farrar@tcd.ie (G.J.F.); 2 The Mater Misericordiae University Hospital, Dublin 7, Ireland; kirkstephenson@hotmail.com (K.S.); dkeegan@mater.ie (D.K.); 3 The Research Foundation, Royal Victoria Eye and Ear Hospital, Dublin 2, Ireland; wynnenc@tcd.ie; 4 Department of Ophthalmology, The Royal Victoria Hospital, Belfast BT12 6BA, Northern Ireland, UK; julie.silvestri@belfasttrust.hscni.net; 5 Centre for Experimental Medicine, Queen’s University Belfast, Belfast BT7 1NN, Northern Ireland, UK

**Keywords:** retina, genetics, ophthalmology, retinitis pigmentosa, genomics

## Abstract

There are an estimated 5000 people in Ireland who currently have an inherited retinal degeneration (IRD). It is the goal of this study, through genetic diagnosis, to better enable these 5000 individuals to obtain a clearer understanding of their condition and improved access to potentially applicable therapies. Here we show the current findings of a target capture next-generation sequencing study of over 750 patients from over 520 pedigrees currently situated in Ireland. We also demonstrate how processes can be implemented to retrospectively analyse patient datasets for the detection of structural variants in previously obtained sequencing reads. Pathogenic or likely pathogenic mutations were detected in 68% of pedigrees tested. We report nearly 30 novel mutations including three large structural variants. The population statistics related to our findings are presented by condition and credited to their respective candidate gene mutations. Rediagnosis rates of clinical phenotypes after genotyping are discussed. Possible causes of failure to detect a candidate mutation are evaluated. Future elements of this project, with a specific emphasis on structural variants and non-coding pathogenic variants, are expected to increase detection rates further and thereby produce an even more comprehensive representation of the genetic landscape of IRDs in Ireland.

## 1. Introduction

Inherited retinal degenerations (IRDs) represent the most frequent cause of vision loss in people of working age. As a result, these conditions have a highly significant impact on quality of life and health-related costs and loss of income. IRDs are an extremely heterogeneous set of conditions associated with the loss of retinal function, and as a group, represent one of the most genetically diverse hereditary conditions. Over 260 genes to date have been implicated in the syndromic and non-syndromic IRDs [1], with a wide range of clinical presentations and rates of progression. As this is a diverse set of conditions with frequently overlapping presentations, it is typically divided into large sub-categories, primarily by specific regions or cell types affected, such as rod photoreceptors, cone photoreceptors or, for example, peripheral versus macular regions of the retina. Retinitis Pigmentosa (RP) is the most common form of IRD, is extremely genetically heterogeneous and affects as many as 1 in 3000 individuals [2,3]. The disease is typically characterized by progressive loss of rod photoreceptor cells, followed by the gradual death of cone photoreceptors and generally involves characteristic features such as pigmentary deposits in the peripheral retina and attenuation of retinal vessels. In contrast, some other forms of IRD can be extremely rare and have a single gene aetiology; gyrate atrophy, for example, is estimated to affect roughly one in a million people [4].

IRDs are currently thought to affect approximately 2.5 million people globally. The vast majority of these individuals have received a diagnosis based on clinical phenotype alone, rather than a genetic diagnosis, if they have been formally diagnosed at all. Clinical trials are in progress for a number of IRDs, however most such trials require patients to have a known causative mutation to participate. Here we present data from Target 5000, an ongoing next generation sequencing (NGS)-based study, which aims to genetically characterise a large national cohort of IRD patients.

The most common method chosen for IRD genetic screening is targeted NGS. Although whole-exome sequencing offers the potential to locate disease-causing mutations in novel genes, in practice diagnosis rates in whole-exome and targeted-sequencing studies are similar [5], suggesting that the coding regions responsible for the majority of IRDs have been located. Although whole genome analysis has the potential to discover non-coding disease-causing mutations, the difficulty involved with data interpretation and cost associated with the study increase dramatically.

During the course of this study, over 750 individuals from over 520 pedigrees have been sequenced with a targeted NGS panel, focused on exons of 254 IRD-associated genes, in addition to a small number of introns previously reported to harbour splice-altering mutations. Here we present novel mutations primarily from over 200 patients involved in recent recruitment but also resulting from retrospective analysis based on previously recruited patient cohorts [6]. Candidate mutations were detected in over 68% of our analysed pedigrees. This figure includes previously reported pathogenic mutations and numerous novel likely pathogenic variants. Novel variants include large structural variants, point mutations with high predicted pathogenicity, frameshift mutations and splice site mutations. A single pathogenic or likely pathogenic variant was observed in an additional 8% of pedigrees in which the gene in question is known to cause a recessive retinopathy.

## 2. Materials and Methods

### 2.1. Patient Identification and Recruitment

Probands and other family members were primarily assessed at the Research Foundation of the Royal Victoria Eye and Ear Hospital (Dublin, Ireland) and the Mater Misericordiae University Hospital (Dublin, Ireland). With informed consent, best-corrected visual acuity was assessed using revised 2000 Early Treatment Diabetic Retinopathy Study (ETDRS) charts (Precision Vision, La Salle, IL, USA). Colour vision was examined using the Lanthony desaturated D-15 panel (Gulden Ophthalmics, Elkins Park, PA, USA) under standardised lighting conditions. Goldmann perimetry was used to assess the peripheral visual fields to the IV4e, I4e and 04e targets. Full-field electroretinograms were performed according to the International Society for Clinical Electrophysiology of Vision (ISCEV) standards [7] using a Roland Consult RETI-port retiscan (Brandenburg an der Havel, Germany). Fundus colour and autofluorescence photography was performed using a Topcon CRC50DX (Topcon Great Britain Ltd., Berkshire, England) or Optos Daytona (Optos plc, Dunfermline, Scotland). Spectral domain optical coherence tomography was performed using a Cirrus HD-OCT (Carl Zeiss Meditec, Berlin, Germany).

### 2.2. DNA Isolation and Next Generation Sequencing

With informed consent, blood samples were taken from patients after a thorough clinical assessment. DNA was isolated from 2 mL of blood and fragmented for targeted sequencing to an average fragment size of 200–250 base pairs (bp).

Sequencing libraries were generated and target capture was performed with the Nimblegen SeqCap EZ kit (Roche Ireland Ltd., Dublin, Ireland), incorporating the exonic regions of 254 genes implicated in retinopathies (Supplementary Data, Table S1: Full list of genes captured in NGS panel). Captures were executed as per the manufacturer’s instructions. Capture regions also included intronic regions in *CEP290, ABCA4* and *USH2A* that are known to potentially contain pathogenic mutations [8,9,10]. The capture panel also included a small number of genes implicated in retinal development and regulation such as *CTBP2*, which encodes the RIBEYE protein, which is essential to the formation of retinal presynaptic ribbons [11]. The total size of the captured region was approximately 750 kb.

Captured patient DNA was initially multiplexed into 24-sample pools using NimbleGen Adapters (Roche Ireland Ltd., Dublin, Ireland) and sequenced using an Illumina MiSeq (Illumina Inc., San Diego, CA, USA). In more recent captures, samples were multiplexed into 96-sample pools using dual-indexed adapters from IDT (Integrated DNA technologies, https://www.idtdna.com/) designed to be compatible with Illumina systems, to enable up to 96-plex pooling for higher-throughput instruments. Confirmatory single-read sequencing was also performed to verify the presence of candidate mutations.

### 2.3. Polymerase Chain Reaction and Sanger Sequencing

In order to validate candidate mutations found in NGS experiments, amplicons containing the mutations were generated by polymerase chain reaction (PCR) and analysed by direct sequencing. Oligonucleotides were purchased from Sigma-Aldrich (Gillingham, England). The target DNA products were amplified using Q5 High-Fidelity 2× Master Mix (New England Biolabs Inc., Ipswich, MA, USA). The annealing temperature for reactions were optimised for each mutation; all other details were executed as per the supplier’s recommendations. Sanger sequencing was performed by Eurofins Genomics (Ebersberg, Germany).

### 2.4. Gyrate Atrophy Inversion Confirmation

Four primers were designed to cover the two breakpoints of the homozygous inversion in the *OAT* gene, located at NC_000010.11:g.124405527 and NC_000010.11:g.124422152. Primers OAT-1 (forward: 5′-GGTAACCTGGATCCGGAACA-3′) and OAT-2 (reverse: 5′-CTTCTGGGGTAGGACGTTGT-3′) covered the breakpoint in exon 5 of *OAT* and primers OAT-3 (forward: 5′-GCGAGGGGTTTCACATCATC-3′) and OAT-4 (reverse: 5′-GTTGGTGTTTCTCTGGCCTG-3′) targeted the breakpoint upstream of the *OAT* gene. In unaffected *OAT* genes the pairing of primers OAT-1 and OAT-2 would form a product, as would the pairings of primers OAT-3 and OAT-4. However, in the specific case of the homozygous inversion reported here, no product would result from these pairings. Products in this case could only be formed when primers OAT-1 and OAT-3 were paired and likewise with primers OAT-2 and OAT-4, which would not generate any product when unaffected patient DNA was used as a template. This is shown in the Supplementary Materials diagrammatically (Figure S1: An illustration of the PCR strategy designed to detect a large homozygous inversion) and experimentally (Figure S2: Confirmation of an *OAT* inversion using strategic PCR design).

### 2.5. Data Analysis

NGS data was demultiplexed and mapped to the human genome (hg38) using BWA version 0.7.15 [12]. Duplicate reads were flagged using Picard version 2.5.0 [13] and downstream analysis and variant calling performed using Freebayes version 1.1.0 [14].

Patient data from earlier stages of this study [6] that has been previously analysed with different software tools and aligned to previous versions of the human reference genome was retrospectively reanalysed using the current updated methods and reference databases for the purpose of expanding mutation detection and consistent reporting. The American College of Medical Genetics and Genomics (ACMG) criteria for classifying pathogenic variants was utilised [15].

Variants were annotated using SnpEff [16], dbNSFP [17], MetaLR [18] and M-CAP [19] for the purposes of identifying rare, pathogenic coding changes. Annotations from the SPIDEX database were used to identify variants with predicted splicing impacts. Common variants, as measured by either frequency within our sample pool or frequency in external population databases, were filtered out of the analysis. Synonymous variants were also filtered out of downstream analysis, with the exception of analysis of potential splice site alterations. 15× coverage was required at a site in order to call variants. This was calculated at the individual base level rather than the exon level. For almost all samples, more than 98% of targeted sites were covered to this depth (interquartile range: 98.3–99.1%).

In addition, structural variants were called by a separate pipeline, based on the tools LUMPY [20], which was used to identify structural variants via split reads or unusual paired read alignments, and CoNIFER [21], which was used to identify structural variants based on aberrant read depths. Results from these tools were combined and filtered to output a final, high-quality list of putative structural variants and associated confidence scores for each sample.

### 2.6. Ethical Approval

Ethical approval for this study was awarded by the Research and Medical Ethics committee of the Royal Victoria Eye and Ear Hospital (13-06-2011: HRA-POR201097) and by the Institutional Review Board of the Mater Misericordiae University Hospital and Mater Private Hospital (MMUH IRB 1/378/1358), Dublin, Ireland prior to commencement. All work was carried out in accordance with the approved guidelines. All patients have given written informed consent before recruitment to the study. No patients under 18 years of age were included in the study.

## 3. Results

### 3.1. Presentation and Solve Rates

**Figure 1:**
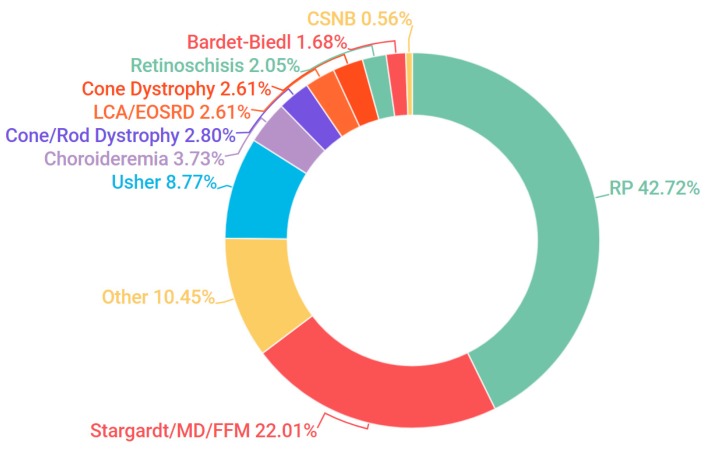
Clinical presentation of all inherited retinal degeneration(IRD) pedigrees included in the study. Abbreviations are listed clockwise as they appear above. RP: retinitis pigmentosa; Stargardt: Stargardt disease; MD: macular dystrophy; FFM: fundus flavimaculatus; Usher: Usher Syndrome; LCA: Leber congenital amaurosis; EOSRD: early-onset severe retinal dystrophy; Bardet-Biedl: Bardet-Biedl syndrome; CSNB: congenital stationary night blindness.

**Figure 2:**
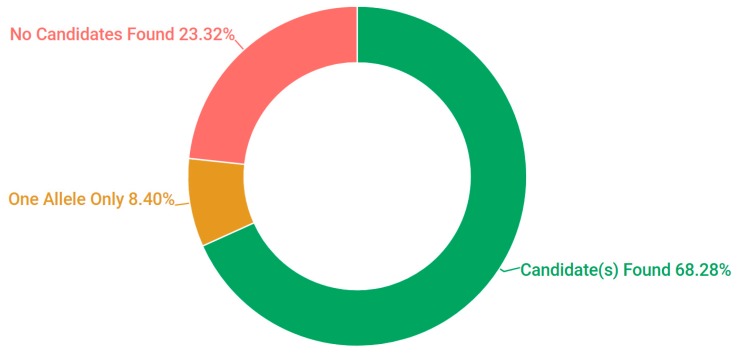
Pedigree solve rates employing target capture next generation sequencing for 254 IRD genes.

Probands, in addition to additional family members where available, were sampled from 523 pedigrees in Ireland. The criteria for inclusion in the study included being over 18 years of age and a full clinical examination that implicated an IRD as a likely cause of visual impairment. A chart depicting the frequency of each condition at clinical presentation is represented in Figure 1. A current list of genes included as part of the targeted sequencing has been provided in the Supplementary Materials (Table S1: Full list of genes captured in NGS panel). A pathogenic or likely pathogenic variant has been detected in over 68% of pedigrees analysed. In addition, in 8% of cases one single candidate mutation could be detected and the gene in question has previously been associated with a recessively inherited IRD; it is likely that at least some of these patients will carry a second mutation in this gene that was not detected, such as a deep intronic or structural variant [22]. This information is further depicted in Figure 2. A large number of likely pathogenic and novel mutations have also been found as part of this study (Table 1).

### 3.2. Stargardt Disease

Stargardt disease (STGD1; OMIM #248200) is the most common inherited macular dystrophy with an estimated incidence ranging from one in 8000 to one in 10,000 [23]. Typical clinical features include bilateral central vision loss with macular atrophy and flecks in the retinal pigmentary epithelium. The age of onset and severity is dependent on the intrinsic pathogenicity of the causative mutations however most disease appears evident within the first two decades of life [24].

Stargardt disease is the second most common IRD observed in our study. *ABCA4* was unsurprisingly the most frequently observed gene associated with STGD1 or its phenotypically similar counterpart, fundus flavimaculatus. *ABCA4* was deemed the candidate gene in a total of 80 pedigrees across these two conditions. Several novel likely pathogenic mutations were observed in this study relating to STGD1, NM_000350.2: c.5917delG, p.Val1973fs; c.735T>G, p.Tyr245*; c.4320delT, p.Phe1440fs. These novel *ABCA4* mutations were all observed in separate Stargardt pedigrees, segregating with the condition in each family and in *trans* with known pathogenic *ABCA4* mutations.

Despite the relative genetic homogeneity of STGD1, *ABCA4* still represents a challenge in terms of diagnostics due to the suspected prevalence of many deep intronic pathogenic mutations [25]. However, as many of these IRD diagnostic studies progress it is likely that the increasing body of data will make it possible to more accurately estimate the pathogenicity of variants discovered during sequencing. One example of this is the reclassification of the hypomorphic *ABCA4* variant p.Asn1868Ile (c.5603A>T). This variant was previously dismissed as benign due to its background population frequency of 7%. This p.Asn1868Ile variant was observed in 94 sequenced patients, including 4 patients who were homozygous for the mutation [26]. The inclusion of this single variant in the analysis has aided in the genetic diagnosis of an additional 19 patients in our IRD patient cohort. The majority of these 19 patients were clinically diagnosed with a milder form of STGD1 or a late-onset macular degeneration but only a single pathogenic or likely pathogenic *ABCA4* mutation could previously be detected. However subsequent to modification of variant calling filters to allow for the inclusion of the p.Asn1868Ile variant, these 19 cases could be solved. The observation in the current study that the p.Asn1868Ile variant is associated with a clinically distinguishable milder form of STGD1 is consistent with the associated phenotypes outlined by Zernant and colleagues [26].

**Figure 3:**
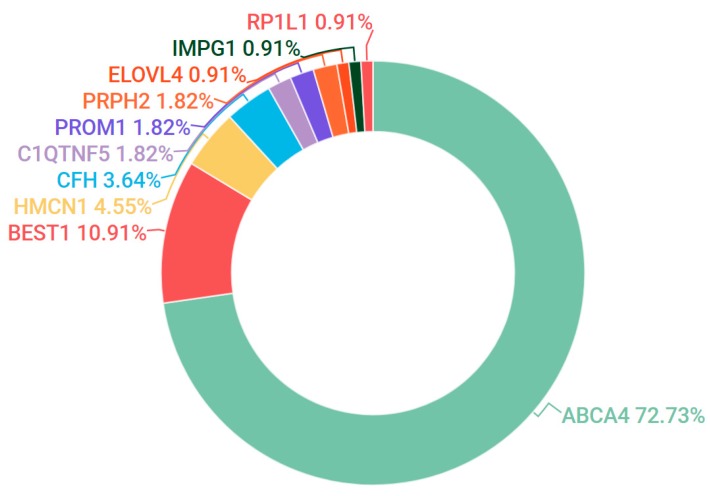
Gene candidates (and percentages) observed in the IRD grouping encompassing Stargardt disease, fundus flavimaculatus (FFM) and macular dystrophy (MD).

*ELOVL4* and *PROM1* also contributed to a small number of pedigrees that presented as Stargardt-like dominant macular dystrophies. Novel likely pathogenic mutations were detected in *ELOVL4* during cohort analysis (Table 1). However, the most common candidate mutations associated with dominant maculopathy in our participants were found in the *BEST1* gene (Figure 3).

### 3.3. Retinitis Pigmentosa

Retinitis Pigmentosa (RP; OMIM #268000) was the most common clinical diagnosis for participants in the current study, accounting for nearly 40% of total pedigrees sequenced. This figure encompasses the various modes of inheritance (autosomal dominant, autosomal recessive and X-linked) and phenotypes (typical, inverse and paravenous among others) associated with RP.

The most common gene candidate found for dominant RP in the study was *RHO* (OMIM #180380). This accounts for over 12% of all RP pedigrees involved in the study and almost 30% of pedigrees diagnosed with dominant RP. This figure is an under representation of the prevalence of *RHO*-linked RP in this IRD population, as several pedigrees involved in prior single-gene studies on *RHO* were excluded from this study, as causative mutations had already been established [27,28,29]. The predominant pathogenic mutations observed are NM_000539.3:c.533A>G, p.Tyr178Cys; c.620T>G, p.Met207Arg, however, numerous novel likely pathogenic mutations have also been detected. Those variants found in sufficiently large pedigrees to verify segregation are shown in Table 1. In prior single-gene studies, the *RHO* mutations p.Tyr178Cys, p.Met207Arg and p.Thr94Ile were also commonly observed in the Irish IRD population.

**Figure 4:**
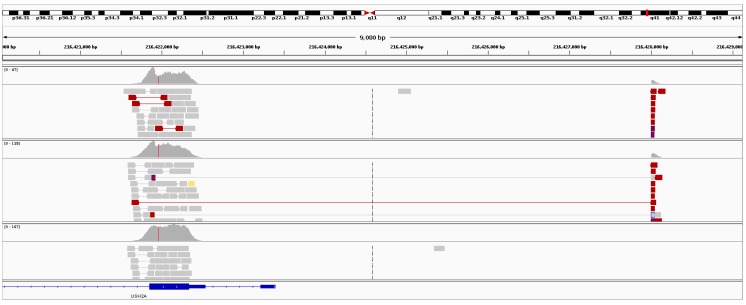
Sequence data from retinitis Pigmentosa (RP) patients with large *USH2A* deletions. The top two panels of reads are from the two affected patients who presented with RP. The bottom panel of reads is from an unaffected unrelated patient. Depicted above is the evidence of split reads (red) and a drop in read depth in the affected patients relative to a control.

*USH2A* (OMIM #608400) was the most frequently observed gene candidate for recessive RP. Pathogenic or novel mutations in this gene were observed in over 30% of recessive RP resolved cases. Although many *USH2A* pathogenic mutations are associated with Usher syndrome, none of the pedigrees which were initially clinically diagnosed with recessive RP showed any other syndromic traits. This finding of non-syndromic retinal degenerations associated with mutations in *USH2A* is consistent with observations based on other *USH2A* cohorts [30]. In one Irish IRD pedigree with two affected individuals diagnosed with recessive RP, a large heterozygous deletion was detected in *USH2A* (Figure 4) employing the structural variants analysis pipeline described in the Methods section. This deletion was approximately 9 kb in size and spans the first 150 amino acids of the coding region, genomic coordinates (hg38): g.chr1:216421919-216428002. This deletion encompasses a significant part of USH2A, including the start codon, transcriptional start site and part of the first exon. As such, it is highly likely to cause complete loss of function. The 9 kb mutation in *USH2A* was detected alongside a reported pathogenic mutation further downstream in the gene in both affected patients in this pedigree, NM_206933.2:c.2276G>T, p.Cys759Phe. An unaffected sibling from this pedigree was directly sequenced for both mutations and was found to be negative for the large deletion (Supplementary Data, Figure S3: Gel confirmation of *USH2A* deletion ) and positive for the pathogenic single base mutation, p.Cys759Phe (Supplementary Data, Figure S4: Sanger sequencing trace of *USH2A* mutation, p.Cys759Phe) confirming that the two *USH2A* mutations observed in this pedigree are found on different alleles. The region surrounding the breakpoint in exon 2 was a long pyrimidine run, encoding a series of serine and proline residues. The region surrounding the breakpoint upstream of USH2A was very similar, although one purine nucleotide was present in the surrounding 25 bp. It is plausible that these sequence similarities facilitated the formation of the deletion.

*RP1* (OMIM #603937) mutations have been found in both dominantly and recessively inherited forms of RP. *RP1*-RP was first described as a dominantly inherited condition [31] and then subsequently found to also be associated with a recessive mode of inheritance several years later [32]. Although there are quite a number of missense pathogenic mutations associated with *RP1*-RP [33], only a single missense mutation was observed in the current IRD cohort, p.Thr373Ile. This *RP1* mutation was found heterozygously in 34 different IRD patients from our study. As this mutation has only been observed to be causative of an IRD when homozygous or as a compound heterozygote [32], it could not be deemed causative in any of the cases in this current study as a second pathogenic mutation was not detected in the *RP1* exonic sequence. All other candidate recessive or dominant mutations in *RP1*-RP deemed to be likely causative of the observed phenotype were frameshift mutations, including a novel recessive mutation, NM_006269.1:c.160delG, p.Val54fs (see Table 1).

**Figure 5:**
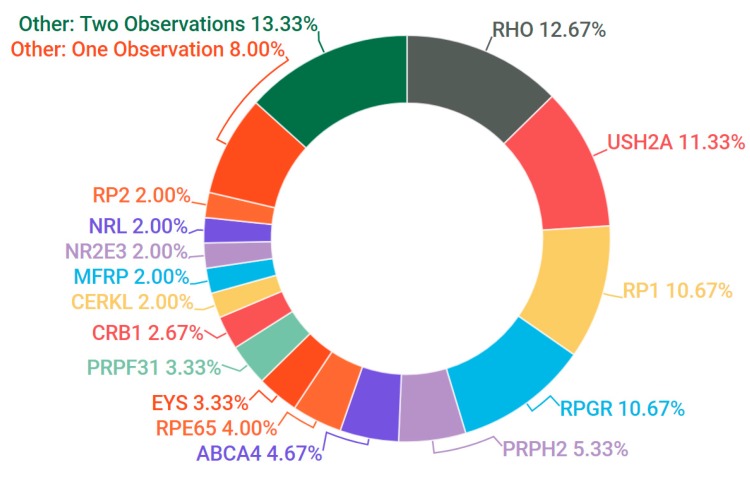
Gene candidates (and percentages) observed in cases of RP. Genes with two observations: *CRX, IFT140, KLHL7, PDE6A, PDE6B, PROM1, PRPF8, ROM1, SLC24A1, SNRNP200*. Genes with single observations: *AHI1, C2orf71, CNGA1, CNGB1, GNAT1, GUCA1B, HK1, IMPDH1, OFD1, PRPF6, RDH12, TULP1.*

Of the X-linked RP cases in which a candidate variant was detected, 80% carried known or likely pathogenic variants in the *RPGR* gene (OMIM #312610). The majority of these causative *RPGR* mutations were frameshift mutations, including one novel mutation, NM_001034853.1: c.2236_2237delGA, p.Glu746fs. Given that the repetitive region of *RPGR*’s orf15 tends to negatively impact NGS efficacy [34], pathogenic mutations were rarely called in this region unless both read quality and depth were of a high standard. A small number of pedigrees also tested positive for candidate mutations in *RP2* (OMIM #300757), including a novel frameshift mutation, NM_006915.2:c.425delA, p.Asn142fs. All candidate genes found in RP pedigrees as part of this study are depicted in Figure 5.

### 3.4. Bardet-Biedl Syndrome

Although only 8 pedigrees in this study were initially clinically diagnosed with Bardet-Biedl Syndrome (BBS; OMIM #209900), an additional 19 pedigrees were rediagnosed as BBS, the majority of which presented clinically as simplex RP as no other syndromic signs characteristic of BBS were obvious to the clinical staff initially. This rediagnosis was prompted by genetic findings and where possible, confirmed by the ophthalmologists associated with the project. A number of these cases were subsequently found to have had polydactyly, which had typically been surgically removed at a young age. Additionally, a smaller number of patients were seen to have more prominent intellectual disabilities and indications of obesity upon annual clinical check-ups. The most frequent mutation detected in the patient population was *BBS1* (OMIM #209901) NM_024649.4:c.1169T>G, p.Met390Arg. This mutation was observed homozygously in 17 pedigrees, and heterozygously in another 4 pedigrees. It was also the most likely BBS mutation to present as simplex RP. The findings are consistent with other studies carried out with Northern European IRD patient cohorts [35,36]. Five additional BBS pedigrees revealed candidate mutations across four other known BBS genes: *BBS4* (OMIM #600374), *BBS9/PTHB1* (OMIM #607968), *BBS10* (OMIM #610148) and *BBS16/SDCCAG8* (OMIM #613524).

### 3.5. Usher Syndrome

**Figure 6:**
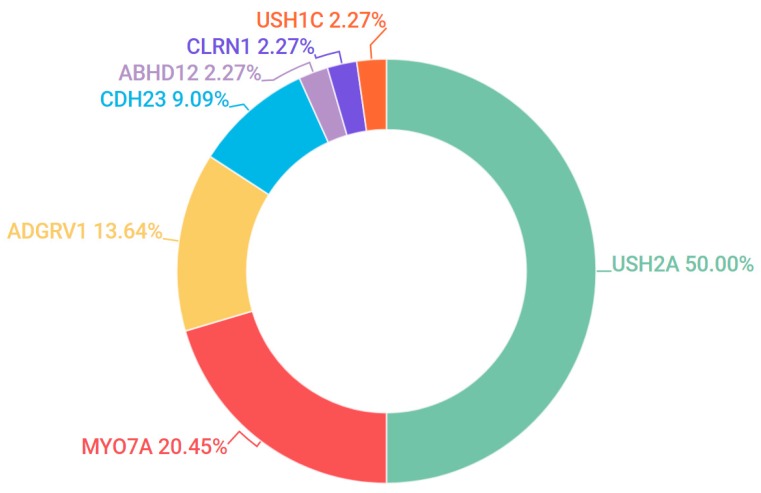
Gene candidates (and percentages) found across all subtypes of Usher Syndrome.

Usher Syndrome (USH) is diagnosed by the concurrent incidence of auditory impairment and RP. Three subtypes of USH were detected in our patient cohort, Type 1 (OMIM #276900), Type 2 (OMIM #276901) and Type 3 (OMIM #276902). These subtypes can be broadly identified by severity of hearing loss: congenital deafness, loss of hearing and progressive loss of hearing respectively. 47 pedigrees in the study were clinically diagnosed with some form of USH and candidate mutations were found in 44 pedigrees (94%). Pathogenic or likely pathogenic candidate mutations in *USH2A* were detected in 50% of all USH cases, which accounts for the majority of USH Type 2 cases. The most frequent candidate gene for USH Type 1 was *MYO7A* (OMIM #276903). Together, *USH2A* and *MYO7A* accounted for over 70% of the pathogenic mutations detected across all USH subtypes (Figure 6).

**Figure 7:**
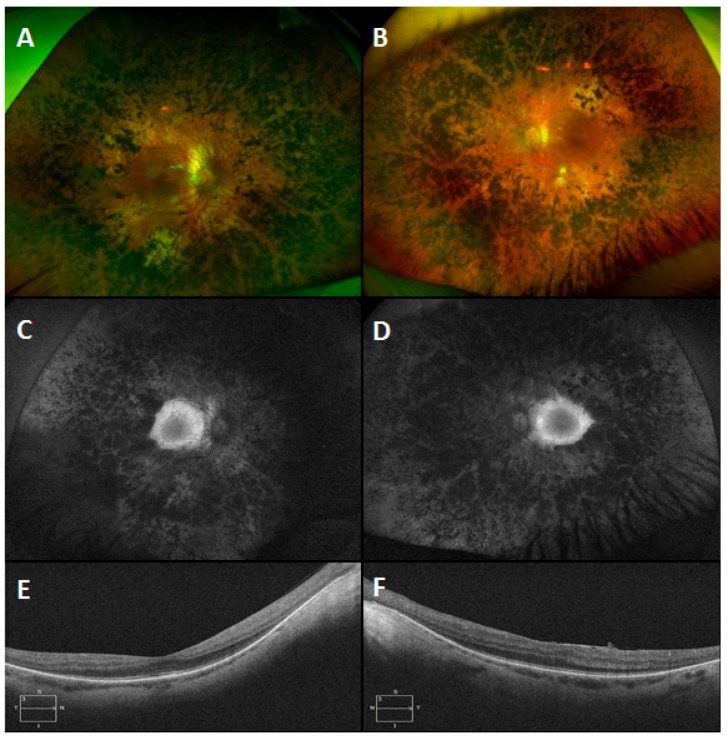
Montage of clinical images from an Usher syndromepatient with a large homozygous deletion in *USH1C*. Colour images of the right (**A**) and left (**B**) eyes of a 44 year old male patient with Usher syndrome type 1 which show symmetrical circumferential mid-peripheral bone spicule pigmentation and RPE atrophy with relative preservation of the maculae. The corresponding autofluorescence images of the right (**C**) and left (**D**) eyes demonstrate peripheral hypoautofluorescence and preserved macular autofluorescence. Note the symmetrical hyperautofluorescent rings, which correspond to the extent of the preserved macular photoreceptors on the Optical Coherence Tomography (OCT) scans (**E**: right eye and **F**: left eye).

**Figure 8:**
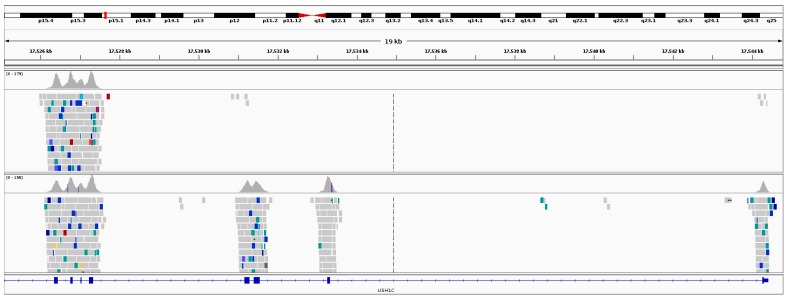
Large homozygous deletion spanning the first four exons of *USH1C*. In the top panel of reads from the patient sample, it is clear that there was no sequenceable template in this region compared to the control (bottom panel of reads).

A large structural variant was also detected in an USH pedigree. In this instance, a patient presented with USH Type 1 (Figure 7) and no variants in USH associated genes were detected. However, once a detailed analysis was undertaken with our structural variants pipeline (Methods: Structural Variants), it became apparent that a large homozygous deletion spanning the first four exons of *USH1C* was present, (Figure 8). These findings were verified by PCR experiments designed to amplify the first four exons of *USH1C* (Supplementary Data, Figure S5: Analysis of *USH1C* deletion by use of PCR products.

### 3.6. Gyrate Atrophy

Gyrate atrophy/gyrate atrophy of choroid and retina (GA/GACR; OMIM #258870) is an extremely rare condition linked with a single gene aetiology, *OAT*. It is estimated that the global incidence of GA is less than one in a million, with the exception of Finland which has an estimated incidence of one in 50,000 [5]. In some regions of the world, such as Australia, the condition is sufficiently rare that the first case of GA has only been reported in the last few years [37]. The *OAT* gene encodes a mitochondrial matrix enzyme, ornithine aminotransferase, which is involved in many biologically fundamental pathways such as the urea cycle, biosynthesis of creatine and proline metabolism [38]. GA has a range of consequential phenotypes associated with it, primarily cystoid macular edema. It is for this reason that an accurate diagnosis is essential as these conditions are becoming increasingly treatable, both through dietary restrictions and medical intervention [39].

**Figure 9:**
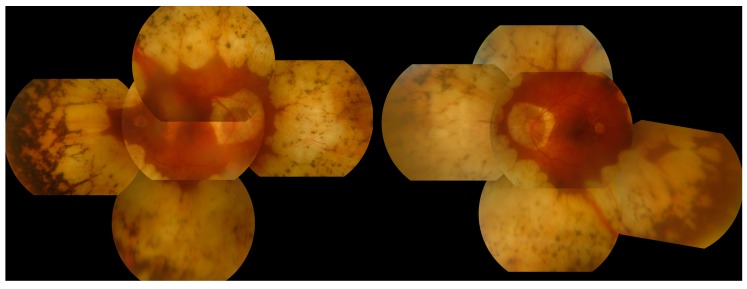
Montage of fundus photography of an affected gyrate atrophy (GA) patient with an *OAT* inversion. Note the marked retinal and choroidal atrophy affecting the mid-peripheral fundus of each eye with relative sparing of the central macular area. The preserved macular areas show the scalloped edges which are characteristic of the condition.

**Figure 10:**
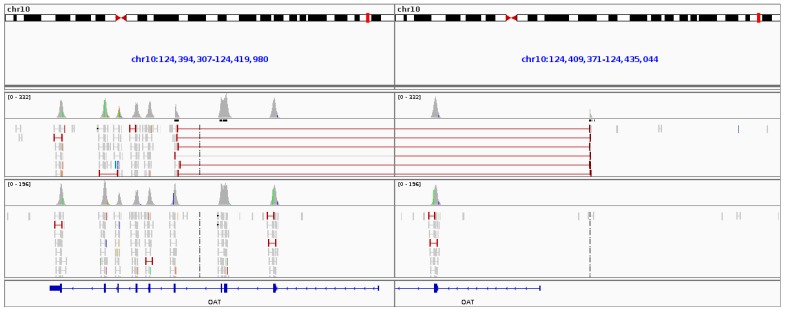
Sequencing results from the gyrate atrophy patient aligned to the reference genome. The top collection of sequence reads are from the affected GA patient and the bottom section of reads are from an unaffected patient sequenced in parallel. Split reads (red) from the affected patient can be seen to be spanning a 16.6 kb region of the *OAT* gene as viewed in IGV (Integrative Genomics Viewer).

**Figure 11:**
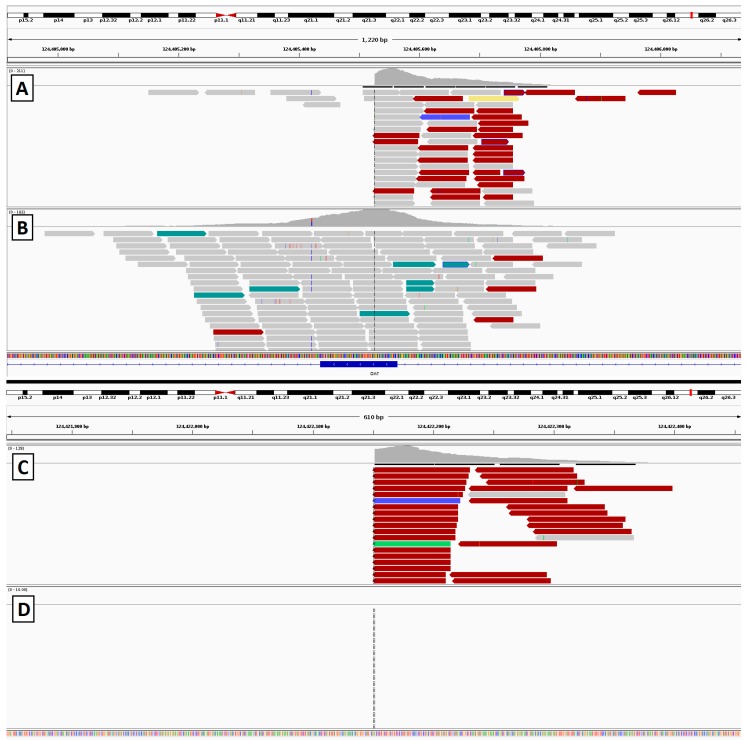
Sequencing results from the gyrate atrophy patient depicting alignment of reads at each breakpoint. (**A**,**B**) show reads surrounding the exonic breakpoint in the affected patient (**A**) and an unaffected control (**B**). (**C**,**D**) show the distal breakpoint. This region is not covered by our capture panel, but in the affected patient sample (**C**) alignments can be seen from library fragments that span the inversion breakpoint. In the control patient (**D**), no aligned reads are seen, as expected.

Here we report a novel homozygous inversion of the *OAT* gene. To the best of our knowledge, this is the first large inversion to be reported in this gene. The patient presented in the clinic with an obvious GA phenotype (Figure 9) and following analysis with the in-house structural variant calling pipeline (Methods: Structural Variants) it was apparent from the breakpoints that a 16.6 kb region had been inverted. The region starts approximately 3 kb upstream of the gene and ends in the middle of exon 5 of the gene (Figure 10 and Figure 11). These findings were subsequently confirmed by alternating primer pairs designed to target the breakpoint regions of this inversion with unaffected patient DNA used as controls (Supplementary Data: Figure S2: Confirmation of an OAT inversion using strategic PCR design).

### 3.7. Novel Variants

Several novel likely pathogenic variants were detected in the course of this study across all conditions. Pathogenicity of novel missense mutations was predicted using the various bioinformatics tools outlined in the Methods section including MetaLR, M-CAP and SPIDEX. Segregation analysis was undertaken for pedigrees sufficiently large to do so where available. A number of the variants reported here (Table 1) had already been allocated a dbSNP ID (*CNGA3* p.Arg410Trp, rs137852608; *RS1* c.326+1G>A, rs281865346) as they had been detected in previous sequencing studies, most likely as part of population studies not specifically focused on the detection of IRDs. Furthermore, those variants that had been previously identified contained no record of known pathogenicity or of the phenotype of the individual in which they were identified.

## 4. Discussion

To date, as part of Target 5000, over 15% of the Irish IRD patient population has been sequenced, providing the first national-scale overview of the IRD landscape. The study offers not only a chance to discover new pathogenic variants in known IRD genes, but represents a vital initial step in the genetic characterisation of patients to provide them with information regarding the underlying genetic pathogenesis of their disease. Previously, we have reported the identification of over 40 novel variants in a smaller cohort of IRD patients [14,40] and here we describe an additional 23 novel mutations and 3 novel structural variants, totaling nearly 70 novel IRD mutations discovered as part of this study. Several mutations that have been previously reported such as *RHO*, p.Met207Arg [28], have presented in multiple pedigrees in this study. It is likely that some or all of these pedigrees are distantly related and current analysis is ongoing to verify this.

Significantly, the genetic pathogenesis of some previously ambiguous disease phenotypes has also been resolved, most notably the milder, late-onset phenotype of Stargardt disease that is associated with the p.Asn1868Ile *ABCA4* mutation. Also, NGS-based genetic diagnoses of IRD patients in this cohort prompted a clinical re-evaluation for many patients, predominantly from simplex RP to BBS, caused by the p.Met390Arg mutation in the *BBS1* gene, patients often presenting with subtle additional phenotypes due to, for example, early intervention for polydactyly.

Many challenges still remain for the application of NGS technologies in diagnostic medicine. Ambiguous disease phenotypes and the presence of disease genes that may be associated with multiple IRDs and different modes of inheritance can make achieving a robust diagnosis particularly difficult. The presence of stretches of repetitive sequence in some IRD genes can also make it difficult to confidently call variants in relevant portions of the genome, which we anticipate may mask some disease-causing variants from analysis. For example, an approximately 800 bp region in the centre of *RPGR* ORF15 shows a sharp drop in mapped reads due to the repetitive nature of the sequence [34]. Given that ORF15 has been implicated in cases of X-linked RP in the past [41], we anticipate that some undiagnosed patients with X-linked retinitis pigmentosa are likely to harbour mutations in this region of the gene, and efforts are underway to augment the protocol we employ to improve coverage of this region; in future sequencing panels, we hope to incorporate an augmented protocol that enhances the success of sequencing in this region. Previous studies have shown that processes can be implemented prior to sample preparation for sequencing [42] or as a parallel investigation [43] to aid sequencing this region.

The RP-associated *RP1* gene also presents some challenges in relation to genetic diagnostics. The pathogenicity and mode of inheritance of a novel mutation in *RP1* is difficult to determine as mutations in this gene have previously been associated with both dominant and recessively inherited disease. This is an issue that has been discussed in a number of other *RP1* studies [44,45,46]. In a recent study, a meta-analysis of previously reported *RP1* pathogenic mutations was undertaken to link the impact of each variant to the functional region of the protein. Difficulties remain in identifying new *RP1* mutations as dominant- or recessive-acting, however, as regions of the gene predominately associated with dominant RP were found to also harbour mutations associated with recessive rod-cone dystrophies [47].

Methods for NGS data analysis are undoubtedly evolving quite rapidly. Research into the effects of splice site mutations and their respective functional impacts is already providing significant insights into the effect(s) of previous potentially overlooked variants in IRD datasets [48,49,50]. Additionally, it is becoming increasingly commonplace for NGS studies of IRD populations to incorporate some form of detection or analysis of copy number variants (CNVs) [51,52]. It has been shown in these studies that close to 20% of previously unsolved IRD pedigrees can be resolved with the detection of pathogenic CNVs.

In the current study we describe the bioinformatics methodologies employed to retrospectively analyse datasets to detect CNVs and sequence breakpoints that are present within the captured exonic regions assessed by target capture NGS. Adopting these methodologies, we have identified three IRD pedigrees carrying three separate large structural variants; a heterozygous large deletion in the *USH2A* gene, a homozygous large deletion in the *USH1C* gene and a homozygous large inversion in the *OAT* gene. The structural variants observed using this approach were identified in genes as diverse as the conditions themselves involving gyrate atrophy (*OAT*), retinitis pigmentosa (*USH2A*) and Usher syndrome (*USH1C*). These findings serve to emphasise the importance of implementing analysis systems that enable detection of large scale deletions and inversions in all IRD patients, as currently, we have observed that 100% of these rare SV events correlate with an IRD gene-associated pathogenic phenotype. Although split-read and read-depth analysis of short-read capture data, as performed in our study, is less sensitive to structural variants than similar methods applied to whole genome sequencing (WGS) data, it has the great advantage that the data it requires is already generated as part of standard sequencing pipelines.

It is highly likely that other structural variants may be present in our cohort but remained undetected as their breakpoints lay outside the exonic regions targeted by the capture panel. This was partly solved by the use of read depth analysis instead, as was successfully applied in the case of the *USH1C* deletion, which had no exonic breakpoints, but this method struggles to detect structural variants that do not span several exons. Despite these limitations, we have demonstrated the utility of this approach for IRD diagnostics by generating clinically actionable results even from past datasets, and we recommend that it be used as a ‘stopgap’ measure to improve diagnosis rates in similar projects before more comprehensive studies can be performed.

Thus far, during the course of the study, genetic analysis of IRD patients has identified candidate mutations in approximately 68% of cases. The diagnostics rates obtained is in line with other NGS studies [4,25,53,54,55]. The growing body of data from NGS studies of IRDs similar to this one should facilitate the formation of better correlations between genotype and phenotype. As research in parallel studies such as natural histories of IRDs [56,57] and functional analysis of modifier loci [49] continue, this information in conjunction with NGS data will undoubtedly contribute to improvements in detecting pathogenic genetic variants responsible for IRDs, as well as providing insights regarding prognoses for some IRDs and importantly may also facilitate the future delivery of gene-specific treatments to the applicable patient populations.

Non-coding variants such as splice-affecting variants, either proximal or distal to canonical splice sites, are also likely to represent a significant fraction of the unobserved disease-causing variants [22,50]. Previous studies have identified deep intronic variants that lead to intronic sequence being incorrectly retained in the mature mRNA as relevant to IRDs [58]. These variants are highly likely to be missed by current studies, as very few capture panels target introns, and the interpretation of deep intronic variants is complicated as these regions are less constrained by purifying selection, leading to large numbers of observed variants. Despite the few direct observations, strong indirect evidence of unobserved disease-causing variants in known IRD genes exists. Whole-exome studies have very similar detection rates to studies focused merely on IRD-associated genes [25], implying that coding mutations in unsequenced genes do not represent a large fraction of unobserved disease-causing mutations. Furthermore, recessive pedigrees that could not be solved in this study with a panel of 254 genes were significantly enriched for single mutations in disease-relevant genes, strongly suggesting the presence of second, as yet unobserved intronic mutations.

Despite these sources of as yet ‘missed’ variation causative of IRDs, the results of this study so far highlight the vast levels of genetic heterogeneity inherent in IRDs in the Irish population and the significant value of a target capture NGS-based genetic evaluation for diagnostic purposes. This has been clearly exemplified by the clinical re-categorisation of the disease pathology for several patients (for example, RP as BBS), the value of detecting pathogenic large structural variants and the continued reanalysis of patient datasets for emerging, previously undetected common pathogenic variants (*ABCA4*, p.Asn1868Ile) all of which were driven by NGS-based genetic data analysis. Future and ongoing studies, with a particular focus on structural variants and non-coding disease-causing variants, are likely to increase mutation detection rates further and yield an even more complete picture of the genetic architecture of IRDs in Ireland.

## Figures and Tables

**Table 1:** Table of Novel Likely Pathogenic Variants. Missense mutations had to show segregation in pedigrees of at least 3 members, two of whom had to be affected. This table does not include the novel large structural variants that are reported in previous sections. The mutations in this table have not been previously associated with IRDs to the best of our knowledge.

Gene	Condition	Transcript ID	Genomic Location	Nucleotide Change	Protein Change	MetaLR Score	Spidex Z-Core	Observed with:
*ABCA4*	Stargardt Disease	NM_000350.2	g.1:94007721	c.5917delG	p.Val1973fs	None	None	p.Arg290Trp
*ABCA4*	Stargardt Disease	NM_000350.2	g.1:94098827	c.735T>G	p.Tyr245 *	None	−3.542	p.Asn1868Ile
*ABCA4*	Stargardt Disease	NM_000350.2	g.1:94030459	c.4320delT	p.Phe1440fs	None	None	p.Asn1868Ile
*ADGRV1*	USH Type II	NM_032119.3	g.1:91153391	c.18795delA	p.Leu6265fs	None	None	p.Arg5772 *
*BBS1*	Bardet-Biedl	NM_024649.4	g.11:66531658	c.1614delC	p.Leu539fs	None	None	p.Met390Arg
*CNGA3*	Cone Dystrophy	NM_001298.2	g.2:98396398	c.1228C>T	p.Arg410Trp	0.9441	None	p.Arg410Trp
*ELOVL4*	Stargardt Disease	NM_022726.3	g.6:79916758	c.789delTAACTTinsAACT	p.Phe265fs_Asn264fs	None	None	/
*MYO7A*	USH Type I	NM_000260.3	g.11:77202351	c.5095C>T	p.Gln1699 *	None	−3.017	p.Ala2009fs
*MYO7A*	USH Type I	NM_000260.3	g.11:77208775	c.6025delG	p.Ala2009fs	None	None	p.Gln1699 *
*NR2E3*	Retinitis Pigmentosa (Recessive)	NM_014249.3	g.15:71813635	c.994G>T	p.Glu332 *	None	None	p.119-2A>C
*NYX*	Congenital Stationary Night Blindness	NM_022567.2	g.X:41474475	c.1022A>C	p.Asp341Ala	0.7224	None	/
*OPA1*	Optic Atrophy	NM_130837.2	g.3:193614740	c.53_62delTGAAACACAG	p.Val18fs	None	None	/
*PRPF31*	Retinitis Pigmentosa (Dominant)	NM_015629.3	g.19:54123748	c.528-1G>T	/	None	−2.131	/
*PRPF8*	Retinitis Pigmentosa (Dominant)	NM_006445.3.2	g.17:1653571	c.6337_6339delAAG	p.Lys2113del	None	None	/
*RHO*	Retinitis Pigmentosa (Dominant)	NM_000539.3	g.3:139531026	c.512C>A	p.Pro171Gln	0.4368	0.875	/
*RP1*	Retinitis Pigmentosa (Recessive)	NM_006269.1	g.8:54621124	c.160delG	p.Val54fs	None	None	p.Val54fs
*RP2*	Retinitis Pigmentosa (X-Linked)	NM_006915.2	g.X:46853795	c.425delA	p.Asn142fs	None	None	/
*RPGR*	Retinitis Pigmentosa (X-Linked)	NM_001034853.1	g.X:38286761	c.2236_2237delGA	p.Glu746fs	None	None	/
*RPGRIP1*	Leber Congenital Amaurosis	NM_020366.3	g.14:21328427	c.2899C>T	p.Gln967 *	None	−3.491	p.Gln967 *
*RS1*	Retinoschisis	NM_000330.3	g.X:18644535	c.416delA	p.Gln139fs	None	None	/
*RS1*	Retinoschisis	NM_000330.3	g.X:18647190	c.326+1G>A	/	None	−2.194	/
*USH2A*	Retinitis Pigmentosa (Recessive)	NM_206933.2	g.1:215647520	c.14791+2T>A	/	None	−2.921	p.Pro4090Thr
*USH2A*	USH Type II	NM_206933.2	g.2015680269	c.12172_12172delCTGinsTAAA	p.Leu4058fs	None	None	p.Glu767fs

## Supplementary Materials

The following are available online at www.mdpi.com/2073-4425/8/11/304/s1, Figure S1: An illustration of the PCR strategy designed to detect a large homozygous inversion; Figure S2: Confirmation of an OAT inversion using strategic PCR design; Figure S3: Gel confirmation of USH2A deletion; Figure S4: Sanger sequencing trace of USH2A mutation, p.Cys759Phe; Figure S5. Analysis of USH1C deletion by use of PCR products, Table S1: Full list of genes captured in NGS panel.
