# Supplementary material for: Target 5000: Target Capture Sequencing for Inherited Retinal Degenerations
Source: Genes (Basel). 2017 Nov 3;8(11):304. doi: 10.3390/genes8110304 (PMC5704217; doi:10.3390/genes8110304)
Supplement: Supplementary file 1 [file genes-08-00304-s001.pdf]

Supplementary Materials

Target 5000: Target Capture Sequencing for Inherited Retinal Degenerations

Adrian Dockery [1,*], Kirk Stephenson [2], David Keegan [2], Niamh Wynne [3], Giuliana Silvestri [4,5], Peter Humphries [1], Paul F. Kenna [1,3], Matthew Carrigan [1,†] and G. Jane Farrar [1,†]

Table S1. Full list of genes captured in NGS panel.

**A**	*BBS9*	*CNGB3*	*GNAT1*	**K**	*NEK2*	*PEX1*	*RDH12*	**T**	*WFS1*
ADIPOR1	*BEST1*	*CNNM4*	*GNAT2*	*KCNJ13*	*NEUROD1*	*PEX2*	*RDH5*	*TEAD1*	*WFS1*
ABCA4	**C**	*COL11A1*	*GNB3*	*KCNV2*	*NMNAT1*	*PEX7*	*RGR*	*TIMM8A*	*WHRN*
ABCC6	*C12orf65*	*COL2A1*	*GNPTG*	*KIAA1549*	*NPHP1*	*PGK1*	*RGS9*	*TIMP3*	**Z**
ABHD12	*C1QTNF5*	*COL9A1*	*GPR179*	*KIF11*	*NPHP3*	*PHYH*	*RGS9BP*	*TMEM126A*	*ZNF408*
ACBD5	*C21orf2*	*CRB1*	*GRK1*	*KIZ*	*NPHP4*	*PITPNM3*	*RHO*	*TMEM216*	*ZNF423*
ADAM9	*C2orf71*	*CRX*	*GRM6*	*KLHL7*	*NR2E3*	*PLA2G5*	*RIMS1*	*TMEM237*	*ZNF513*
ADAMTS18	*C8orf37*	*CSPP1*	*GUCA1A*	**L**	*NR2F1*	*PLK4*	*RLBP1*	*TOPORS*	
ADGRA3	*CA4*	*CTBP2*	*GUCA1B*	*LAMA1*	*NRL*	*PNPLA6*	*ROM1*	*TREX1*	
ADGRV1	*CABP4*	*CTNNA1*	*GUCY2D*	*LCA5*	*NYX*	*POC1B*	*RP1*	*TRIM32*	
ADIPOR1	*CACNA1F*	*CYP4V2*	**H**	*LRAT*	**O**	*POMGNT1*	*RP1L1*	*TRNT1*	
AGBL5	*CACNA2D4*	**D**	*HARS*	*LRIT3*	*OAT*	*PRCD*	*RP2*	*TRPM1*	
AHI1	*CAPN5*	*DHDDS*	*HGSNAT*	*LRP5*	*OFD1*	*PRDM13*	*RP9*	*TSPAN12*	
AIPL1	*CC2D2A*	*DHX38*	*HK1*	*LZTFL1*	*OPA1*	*PROM1*	*RPE65*	*TTC8*	
ALMS1	*CDH23*	*DMD*	*HMCN1*	**M**	*OPA3*	*PRPF3*	*RPGR*	*TTLL5*	
ARL2BP	*CDH3*	*DRAM2*	*HMX1*	*MAK*	*OPN1LW*	*PRPF31*	*RPGR*	*TTPA*	
ARL3	*CDHR1*	*DTHD1*	**I**	*MAPKAPK3*	*OPN1MW*	*PRPF4*	*RPGRIP1*	*TUB*	
ARL6	*CEP164*	**E**	*IDH3B*	*MERTK*	*OPN1SW*	*PRPF6*	*RPGRIP1L*	*TUBGCP4*	
ASRGL1	*CEP250*	*EFEMP1*	*IFT140*	*MFN2*	*OTX2*	*PRPF8*	*RS1*	*TUBGCP6*	
ATF6	*CEP290*	*ELOVL4*	*IFT172*	*MFRP*	**P**	*PRPH2*	*RTN4IP1*	*TULP1*	
ATXN7	*CERKL*	*EMC1*	*IFT27*	*MFSD8*	*PANK2*	*PRPS1*	**S**	**U**	
**B**	*CFH*	*EXOSC2*	*IMPDH1*	*MIR204*	*PAX2*	**R**	*SAG*	*UNC119*	
BBIP1	*CHM*	*EYS*	*IMPG1*	*MKKS*	*PCDH15*	*RAB28*	*SDCCAG8*	*USH1C*	
BBS1	*CIB2*	**F**	*IMPG2*	*MKS1*	*PCYT1A*	*RAX2*	*SEMA4A*	*USH1G*	
BBS10	*CLN3*	*FAM161A*	*INPP5E*	*MTTP*	*PDE6A*	*RB1*	*SLC24A1*	*USH2A*	
BBS12	*CLRN1*	*FLVCR1*	*INVS*	*MVK*	*PDE6B*	*RBP3*	*SLC25A46*	**V**	
BBS2	*CLUAP1*	*FSCN2*	*IQCB1*	*MYO7A*	*PDE6C*	*RBP4*	*SLC7A14*	*VCAN*	
BBS4	*CNGA1*	*FZD4*	*ITM2B*	**N**	*PDE6G*	*RCBTB1*	*SNRNP200*	**W**	
BBS5	*CNGA3*	**G**	**J**	*NBAS*	*PDE6H*	*RD3*	*SPATA7*	*WDPCP*	
BBS7	*CNGB1*	*GDF6*	*JAG1*	*NDP*	*PDZD7*	*RDH11*	*SPP2*	*WDR19*	

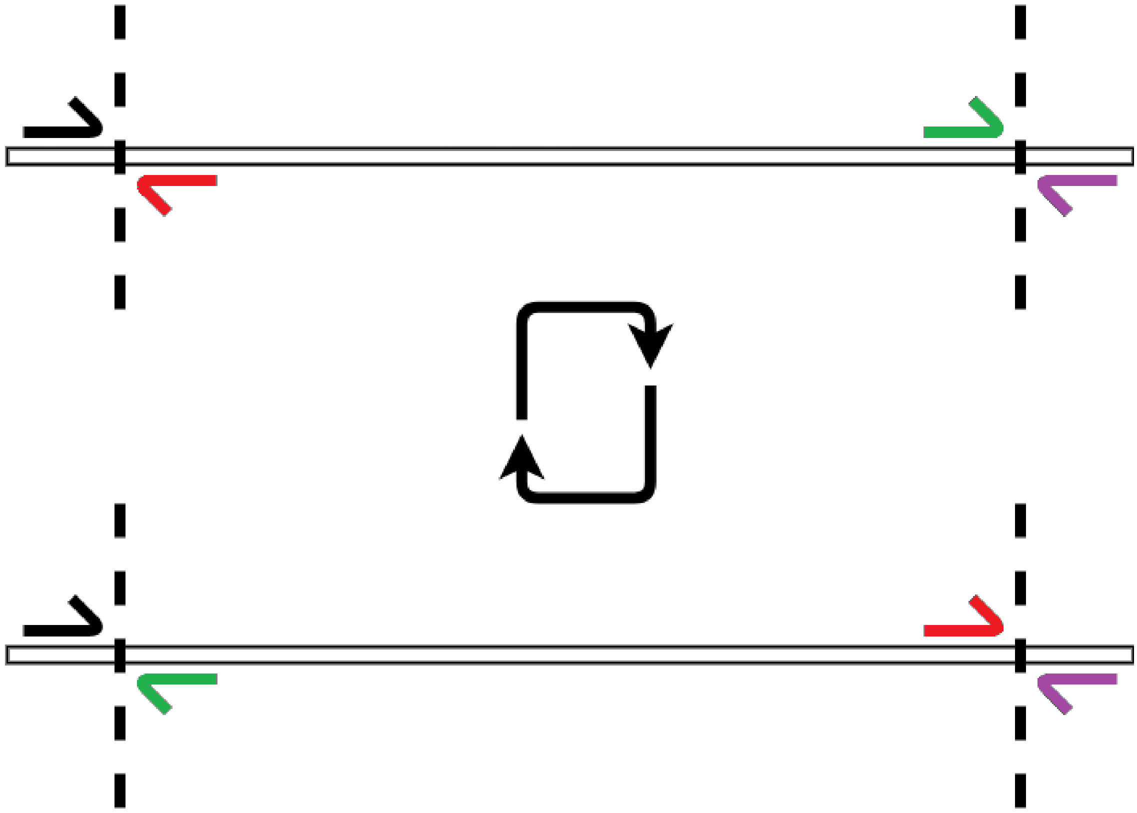

Figure S1. An illustration of the PCR strategy designed to detect a large homozygous inversion. Primers are indicated by colour; OAT-1 (**black**), OAT-2 (**red**), OAT-3 (**green**) and OAT-4 (**purple**). Broken lines indicate genomic breakpoints. Circular arrows indicate an inversion event. The top illustration depicts how primers would anneal around the breakpoints in a control sample. The bottom illustration shows how the internal primers (OAT-2 and OAT-3) are shuffled in an inversion event to form new primer pairings, detectable by PCR analysis.

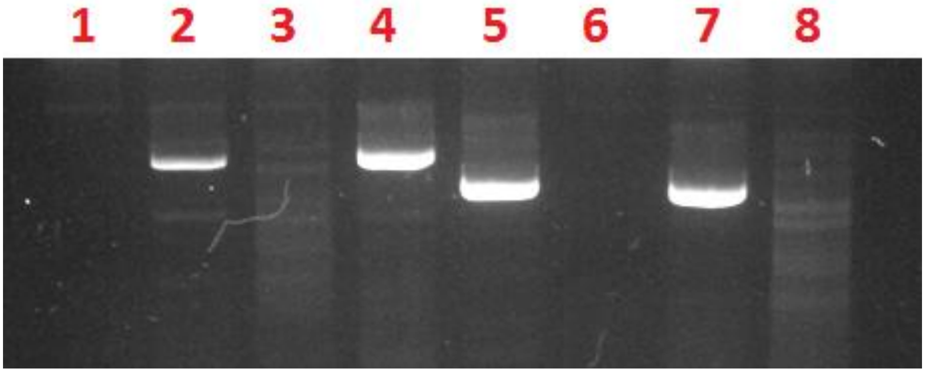

Figure S2. Confirmation of an *OAT* inversion using strategic PCR design. If the wildtype *OAT* sequence is present, the primer sets 1 + 2 and 3 + 4 can be used to generate a PCR product. If the inversion has occurred primer sets 1 + 3 and 2 + 4 can be used to generate of a PCR product. Unaffected patients is positive for products of a wildtype sequence. Patient H58 is positive only for inversion-specific products. Lane 1: Patient H58 Primers 1 and 2; Lane 2: Patient H58 Primers 3 and 4; Lane 3: Patient H58 Primers 1 and 3; Lane 4: Patient H58 Primers 2 and 4; Lane 5: Unaffected Patient Primers 1 and 2; Lane 6: Unaffected Patient Primers 3 and 4; Lane 7: Unaffected Patient Primers 1 and 3; Lane 8: Unaffected Patient Primers 2 and 4.

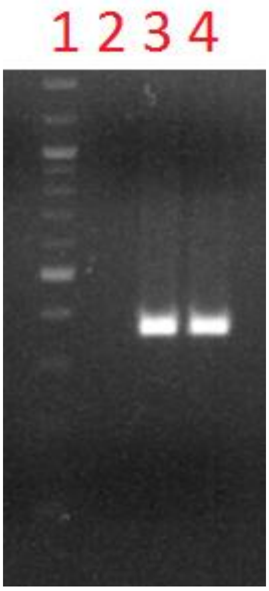

Figure S3. Gel confirmation of *USH2A* deletion. Primers were designed to target the region of deletion in affected patients C41 and E15. A product of the desired size (~300bp) would only be formed if the deletion was present. The sample from the unaffected sibling in the same pedigree, P36, did not form a product. A product was formed from the samples of C41 and E15. Lane 1: 100 bp Ladder; Lane 2: Unaffected Patient P36; Lane 3: Affected Patient C41; Lane 4: Affected Patient E15.

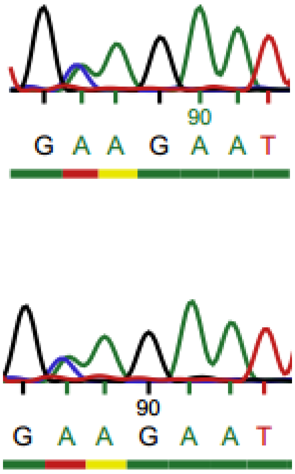

Figure S4. Sanger sequencing trace of *USH2A* mutation, p.Cys759Phe. The top trace is unaffected patient P36 and the bottom trace is affected patient E15. It is clear that both patients share the same heterozygous point mutation.

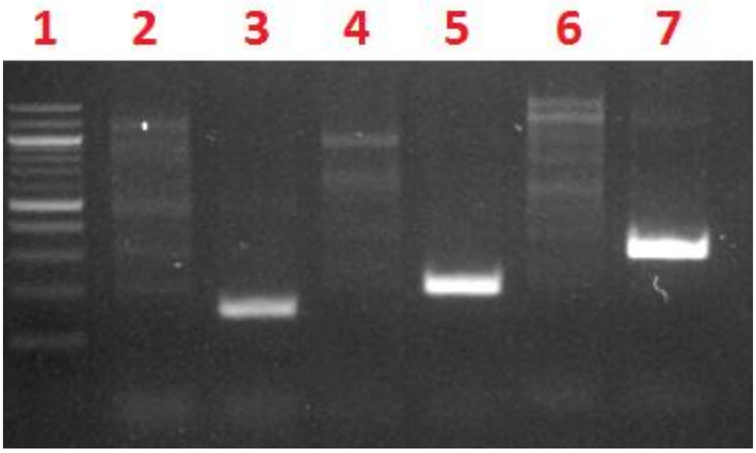

Figure S5. Analysis of *USH1C* deletion by use of PCR products. Primers were designed to amplify each exon believed to be deleted in Patient 1363. If the sequence for the exon was present a product would be formed. Exons 3 and 4 were designed as a single amplicon due to their proximity. The unaffected individual is positive for all of the *USH1C* exons. Patient 1363 is negative for all *USH1C* exons. Lane 1: 100 bp Ladder; Lane 2: Patient 1363 with primers for exon 1; Lane 3: Unaffected Patient with primers for exon 1; Lane 4: Patient 1363 with primers for exon 2; Lane 5: Unaffected Patient with primers for exon 2; Lane 6: Patient 1363 with primers for exons 3 + 4; Lane 7: Unaffected Patient with primers for exons 3 + 4.
